# Defining Digital Public Health and the Role of Digitization, Digitalization, and Digital Transformation: Scoping Review

**DOI:** 10.2196/30399

**Published:** 2021-11-26

**Authors:** Ihoghosa Iyamu, Alice X T Xu, Oralia Gómez-Ramírez, Aidan Ablona, Hsiu-Ju Chang, Geoff Mckee, Mark Gilbert

**Affiliations:** 1 School of Population and Public Health University of British Columbia Vancouver, BC Canada; 2 Clinical Prevention Services British Columbia Centre for Disease Control Vancouver, BC Canada; 3 CIHR Canadian HIV Trials Network Vancouver, BC Canada

**Keywords:** digital public health, digital transformation, digitalization, scoping review, digitization, definition, mobile phone

## Abstract

**Background:**

The recent proliferation and application of digital technologies in public health has spurred interest in *digital public health*. However, as yet, there appears to be a lack of conceptual clarity and consensus on its definition.

**Objective:**

In this scoping review, we seek to assess formal and informal definitions of digital public health in the literature and to understand how these definitions have been conceptualized in relation to digitization, digitalization, and digital transformation.

**Methods:**

We conducted a scoping literature search in Ovid MEDLINE, Embase, Google Scholar, and 14 government and intergovernmental agency websites encompassing 6 geographic regions. Among a total of 409 full articles identified, we reviewed 11 publications that either formally defined digital public health or informally described the integration of digital technologies into public health in relation to digitization, digitalization, and digital transformation, and we conducted a thematic analysis of the identified definitions.

**Results:**

Two explicit definitions of digital public health were identified, each with divergent meanings. The first definition suggested digital public health was a reimagination of public health using new ways of working, blending established public health wisdom with new digital concepts and tools. The second definition highlighted digital public health as an asset to achieve existing public health goals. In relation to public health, *digitization* was used to refer to the technical process of converting analog records to digital data, *digitalization* referred to the integration of digital technologies into public health operations, and *digital transformation* was used to describe a cultural shift that pervasively integrates digital technologies and reorganizes services on the basis of the health needs of the public.

**Conclusions:**

The definition of digital public health remains contested in the literature. Public health researchers and practitioners need to clarify these conceptual definitions to harness opportunities to integrate digital technologies into public health in a way that maximizes their potential to improve public health outcomes.

**International Registered Report Identifier (IRRID):**

RR2-10.2196/preprints.27686

## Introduction

### Background

The past two decades have been characterized by rapid proliferation and application of digital technologies in various domains of public health [1,2]. A wide range of these digital technologies, including mobile apps, social media, wearables, artificial intelligence, and big data, have been deployed with promises of increased speed, efficiency, and cost-effectiveness of public health services [3]. The increasing importance of digital technologies in public health is underscored by the creation of strategic frameworks by international and regional public health agencies to harness the potential benefits of digital technologies to improve public health outcomes [4].

Recognition of the importance of digital technologies in public health services is further emphasized by the increasingly common use of the term *digital public health* within public health discourse [5]. The use of this term was popularized following the publication of the digital-first strategy of Public Health England in 2017 [6]. It has now been used to describe a wide range of public health activities that consider and use digital technologies [6]. For example, universities around the world have begun to offer graduate programs in digital public health [7,8], some public health journals have made calls for special editions on digital public health [9], and others have established topical sections with a specific focus on digital public health [10]. Digital public health conferences and high-level intergovernmental forums on digital public health are also now commonplace [11].

Although the integration of digital technologies into clinical medicine has been broadly characterized within the eHealth and digital health literature, the unique considerations of integrating digital technologies into public health, including services focused on disease prevention and population health needs, have been less well defined [12,13]. The intended meaning of digital public health appears to be implicitly understood as the integration of digital technologies in delivering public health services [7,9]. However, there appears to be a lack of clarity and consensus on a formal definition of digital public health. As interest in the use of digital technologies in public health grows exponentially, especially with recent developments in response to the COVID-19 pandemic [14], the need to clearly define digital public health has become more pressing. Achieving such conceptual clarity and consensus can help inform and align ongoing development, advocacy, policy, research, and implementation in the field to maximize its impact on achieving public health goals, as well as facilitate the evaluation of these efforts across jurisdictions.

### Objective

To the best of our knowledge, there are presently no published reviews aimed at defining digital public health in the literature. Therefore, we aim to understand how public health researchers and practitioners conceptualize and define digital public health through a scoping review of the literature. Given the nascent nature of the field of digital public health, we framed our review to include both formal and informal definitions and descriptions of digital public health found in the existing literature.

## Methods

### Overview

The findings presented in this study are part of a larger scoping review aimed at conceptualizing the breadth of digital public health, the methods for which are described elsewhere [15]. For this study, we used the framework by Arksey and O’Malley [16] with adaptations as suggested by Levac et al [17]. This framework is useful in clarifying complex concepts and has been widely used in emergent fields of health research where available evidence is heterogeneous. Our reporting adheres to the Preferred Reporting Items for Systematic Reviews and Meta-Analyses (PRISMA) guidelines for scoping reviews [18].

### Data Sources

We searched the MEDLINE (Ovid) and Embase (Ovid) bibliographic and citation databases for relevant literature on digital and public health. Gray literature searches were conducted using Google Scholar and manual searches of 14 agency and country-specific websites (Multimedia Appendix 1). Manual reference list searches were also conducted for included articles to identify additional publications of relevance. This expansive literature search was pragmatic given the emergent nature of the field of digital public health.

We explored the intersection between digital health and closely related domains (eg, virtual health, mobile health or mHealth, eHealth, digit* or different suffixes of *digit*, such as digitization) with public health domains as described by the Canadian Public Health Association [19] to determine our final search terms to be applied to the bibliographic databases. This approach balanced our aim to comprehensively assess the literature while ensuring precision by including general search terms such as *digital health* and *public health*. Considering that our focus was on broad conceptual articles, we included search terms (Textbox 1) that excluded primary studies such as cross-sectional studies and clinical trials using the *NOT* Boolean operator. We limited our searches to publications in English and a time frame between January 2000 and June 2020, the time when we conducted the literature search.

**Search terms.**

**Digital health**

“mHealth” or “m-health,” “eHealth” or “e-health,” “virtual health,” “mobile health,” “online health,” “internet-based health,” “computer-based health,” “health informatics,” “social media,” “predictive algorithms,” “artificial intelligence,” “machine learning,” “big data,” “web-based health,” “digital public health,” “digital health,” “digitalization,” “digital tools,” “digital technologies,” “telehealth”

**Public health (combined using AND)**

“public health,” “health promotion,” “health prevention,” “health protection,” “health policy,” “health determinants,” “health evaluation,” “health economics,” “public health ethics,” “risk assessment,” “epidemiology,” “community health,” “emergency preparedness,” “emergency response,” “health equity,” “social justice,” “social determinants,” “public health surveillance”

**Excluded (using NOT)**

“trial,” “cross-sectional”

In addition, we used a simpler search strategy for the gray literature, using only the search terms *digital* AND *public health* on Google Scholar given that traditional advanced search terms as described above yielded imprecise results. The same terms were applied in the Google search engine to inspect 14 preidentified government and intergovernmental agency websites (Multimedia Appendix 1). For example, to search the Government of Canada website [20], we used the search terms *digital* and *public health* with *site:canada.ca* on Google to identify relevant publications. This was done to ensure consistency of our searches across included websites. The first 100 returns from Google Scholar and each website were reviewed. Relevant publications from all searches were exported to Covidence (Veritas Health Innovation) [21] for citation management and review.

### Screening Procedure

**Figure 1:**
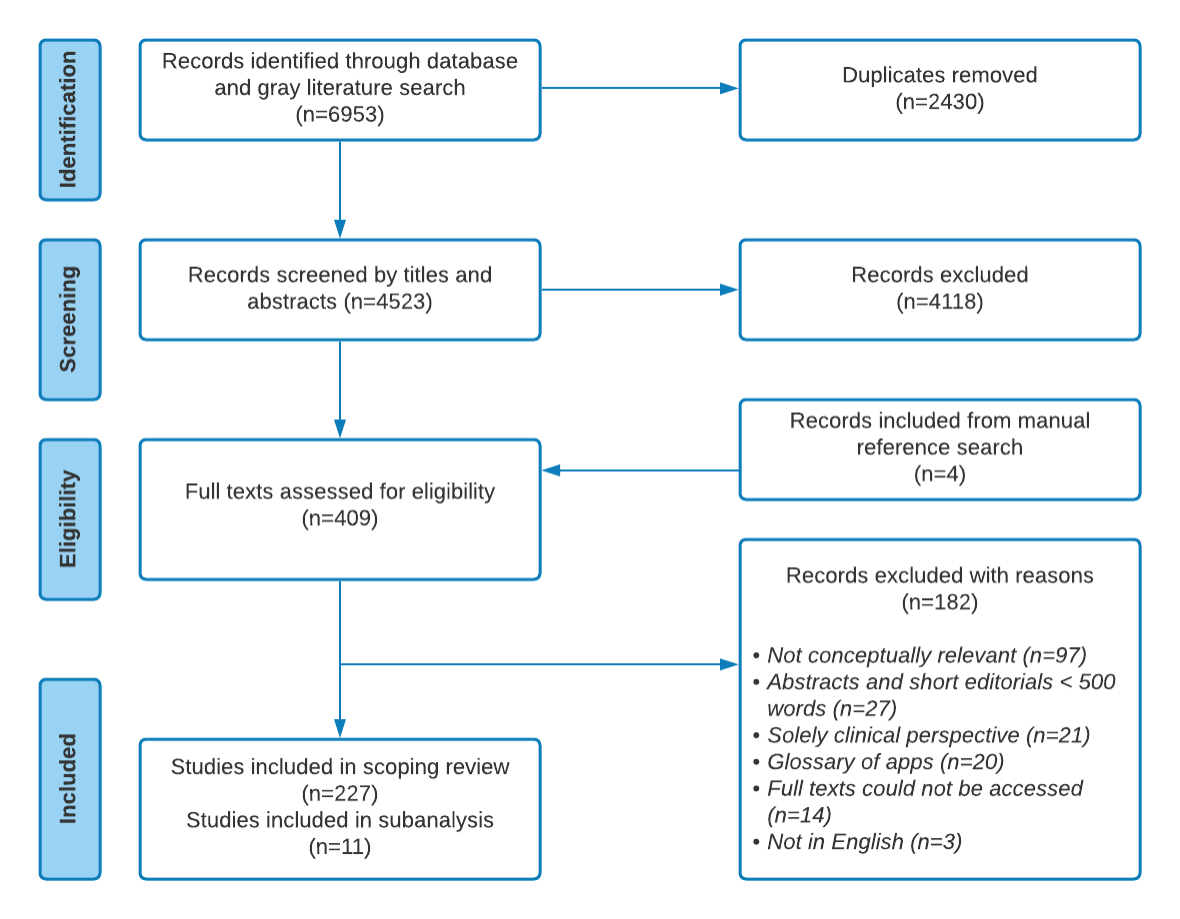
Flow diagram of the search and study selection process following the PRISMA (Preferred Reporting Items for Systematic Reviews and Meta-Analyses) guidelines for scoping reviews.

Titles and abstracts were screened on the basis of pre-established inclusion and exclusion criteria. We included articles that broadly conceptualized digital health from a population and public health perspective and were published in English between January 2000 and June 2020. We drew on the definition of public health by the Canadian Association of Public Health: an organized effort of society to keep persons healthy and prevent injury, illness, and premature death, including a combination of programs, services, and policies that protect and promote the health of all [19]. We excluded publications evaluating specific health programs or interventions and those focused solely on clinical perspectives or short summaries of less than 500 words. Two reviewers (II and AXTX) screened 25% (1131/4523) of the titles and abstracts independently and discussed them to resolve screening discrepancies. The remaining titles and abstracts were screened by at least one reviewer. All full texts and gray literature included in the initial screening were then independently assessed by both reviewers using a structured framework (Multimedia Appendix 2), with discussions to resolve discrepancies and achieve consensus for inclusion. For this analysis of full texts included in the review, we identified articles that formally or informally defined the notion of digital public health. During our review, we found that a significant number of articles made references to the terms *digitization*, *digitalization*, and *digital transformation* in public health. Therefore, we expanded our review to include articles that clarified the roles of digitization, digitalization, and digital transformation in relation to public health. A summary of the selection process is described in Figure 1.

### Data Extraction and Analysis

Selected full texts were imported into QSR NVivo version 12. The bibliographic characteristics of the selected articles, including article type, publication year, country where the research was conducted, and continent of institutional affiliation of the first author, were extracted. The extraction and analysis were conducted by one reviewer, who discussed emergent perspectives and findings with the research team to refine the analysis. Considering that the analysis aimed to derive meanings for digital public health, including both formal and informal definitions of digital public health and related terms, we applied a thematic analysis to the selected papers following the recommendations of Braun and Clarke for thematic analysis [22]. Beginning with initial data familiarization and coding using inductive techniques, we noted references to the integration of digital technologies in relation to public health using the terms *digitization*, *digitalization*, and *digital transformation*. We searched through initial codes to identify substantive definitions and implicit definitions relative to these terms. Finally, we reviewed the themes and summarized the perspectives identified in the form of a narrative report.

## Results

### Overview

The characteristics of the 11 articles included in our review are presented in Table 1. All selected articles were published between 2009 and 2020, with 91% (10/11) published between 2016 and 2020, reflecting the nascent nature of the subject. Furthermore, 82% (9/11) were led by authors with institutional affiliations in Europe, with only 1 article published by an author in North America. All 11 articles, to varying extents, clarified the roles of digitization, digitalization, and digital transformation in relation to public health.

**Table 1:** Characteristics of included articles defining digital public health and clarifying digitization, digitalization, and digital transformation in relation to public health (January 2000 to June 2020; N=11).

Study	Article type	Country or agency of first author	Continent	Definition of digital public health	Description of digitization	Description of digitalization	Description of digital transformation
Azzopardi-Muscat and Sørensen [5] and Azzopardi-Muscat et al [23]	Commentary	Malta	Europe	Not stated	Not stated	Not stated directly; it supposes that digitalization extends beyond platforms and mechanisms, through which patients interact with health services, to include health-related smartphone apps, quantified self-measurements of physiological variables, and use of big data drawing on lifestyle data to profoundly alter health outcomes.	An important and influential process that has substantial impact on health systems and will fundamentally alter the future of health.
Expert Panel on Effective Ways of Investing in Health [24]	Report	European Commission	Europe	Not stated	The process of changing information or data into a digital format. It involves creating a digital version (using bits and bytes) of analog or physical sources such as documents, images, sounds, and more. This creates a code, which can subsequently be used in the context of a process, product, or service. In this case, in a health service.	The use of digital technologies in the context of the production and delivery of a product or service. Such digital technologies allow health services to be organized, produced, and delivered in new ways. It could range from the use of computers and electronic health records to home monitoring of patients, electronic medical devices, and computer-aided visualization.	An important and influential process that has substantial impact. It is a complex and multifaceted issue. It indicates that health care services and systems are in a transition in which more health services and processes will be digitalized. It encompasses the instrumented effort to meaningfully introduce new digital information and communication technologies and the corresponding new processes into the health care sector.
Fitzpatrick et al [25]	Review	Ireland	Europe	Not stated	Not stated	Not stated	Implies cultural process of change that appreciates that one size does not fit all settings.
Lu [26]	Commentary	United States	North America	Not stated	Changing reports so that their contained information may be available electronically.	Not stated	Not stated
Mählmann et al [27]	Commentary	Netherlands	Europe	Not stated	Not stated	Not stated	A driver of change in all industries, through which the collection, storage, processing, and analysis of large amounts of heterogeneous data may occur at high speed.
Odone et al [28]	Commentary	European Public Health Association	Europe	Digital public health is not a discipline per se but an asset the public health community has to fulfill its aims and mission. The health system goals of quality, accessibility, efficiency, and equity of health care embraced by public health professionals are unaltered by the process of digitalization.^a^	Not stated	Digitalization is a set of tools or a means to achieve public health aims and not an aim in itself. It should support and enable the implementation of public health principles but not modify them.	Not stated
Public Health England [6]	Report	England	Europe	A reimagining of public health using new ways of working, blending established public health wisdom with new digital concepts and tools. It recognizes the rapidly changing context of technology, exploring new models of public health using technology and introducing flexibility and resilience that will allow us to adapt our public health practice and improve outcomes.^b^	Not stated	Not stated	End-to-end transformation of public health services founded strongly on user needs. It requires the harnessing and creation of novel, nontraditional partnerships across governments, academia, the technology industry, and scientific bodies. Such transformation can leverage multiple skills and resources to help drive efficiency and deliver value for money across public health.
Rachadell et al [29]	Report	European Public Health Association	Europe	Not stated	A technical process	The use of digital technologies in the context of production and delivery of a product or service. Such digital technologies allow health services to be organized, produced, and delivered in new ways.	A complex but important and influential process that has a substantial impact on health care.
Ricciardi et al [30]	Commentary	Italy	Europe	Not stated	Digitization is a technical process.	The use of digital technologies in the context of the production and delivery of a product or service. Such digital technologies allow health care services to be organized, produced, and delivered in new ways. Digitalization is therefore less of a *technical* process (like digitization), it is also an organizational and cultural process.	Digital transformation encompasses the instrumented effort to meaningfully introduce new digital information and communication technologies and the corresponding new processes into the health care sector. This process may be influenced by ongoing developments outside the health sector.
World Health Organization [4]	Report	World Health Organization	N/A^c^	Not stated	Not stated	Not stated	A disruptive process that allows for the integration of technologies such as the internet of things, artificial intelligence, big data analytics, and blockchain, along with interoperability of patient data through health data standards to potentially enhance health outcomes by advancing disease detection and response, health outcomes by improving medical diagnosis, data-based treatment decisions, and self-management of care.
World Health Organization Regional Office for Europe [31]	Report	World Health Organization	Europe	Not stated; however, the authors note that there is a need to advocate for stronger links between digital health and public and population health objectives and to align the work of digital partners inside and outside the health sector.	Not stated	Digitalization of health systems encompasses the establishment and ongoing maintenance of certain basic elements of infrastructure, including but not limited to hospital information systems, electronic health records and associated clinical support systems, electronic prescription and dispensing systems, telehealth and telemedicine (the provision of health care from a distance), registers and registries, mobile health, public health surveillance, and information portals for patients and health professionals.	Not stated

^a^Predicated on the digital transformation of public health services that is founded on user needs.

^b^Definition predicated on the digitalization of public health practices. Digital tools therefore serve to facilitate the already established public health goals and functions in a way that allows the practice to reap the potential benefits of digitalization.

^c^N/A: not applicable.

### Defining Digital Public Health

Regarding the main objective of our analysis to understand the definition of digital public health, we only found 3 articles that formally offered 2 definitions of digital public health within our sample (Table 1). One of the articles was by Public Health England and defined digital public health as a reimagination of public health, blending established public health wisdom with new digital concepts and tools [6,32]. This article further described digital public health as the exploration of new models of public health using technology while introducing flexibility and resilience to allow the adaptation of public health practice to improve health outcomes [6,32]. However, a second clear description offered by Odone et al [28] referred to digital public health not as a discipline per se but as an asset that the public health community can use to fulfill its aims and mission to ensure quality, accessibility, efficiency, and equity of health care—aims that remain unaltered by the process of digitalization.

### Digitization of Public Health

Textbox 2 summarizes the perspectives identified in the selected articles relating to digitization, digitalization, and digital transformation in public health. In relation to public health, 3 articles described digitization as a *technical process* of converting analog (including paper-based) health records to digital formats that may then be available for use electronically [24,26,30].

**Emergent perspectives on digitization, digitalization, and digital transformation from thematic analysis (January 2000 to June 2020).**

**Digitization**

- Technical process [24,26,30]

**Digitalization**

- Inclusion of technology in producing and delivering services [24,29,30]
- Facilitates new ways of delivering health services [24,29,30]
- An organizational and cultural process [29,30]
- Supports but does not change public health goals [28]
- Ongoing establishment and maintenance of technology for health services [31]

**Digital transformation**

- Complex and multifaceted process [6,24,29,30]
- Fundamental change in the culture and model of service delivery [24,25,30]
- Ongoing change process [24,29,30]
- A disruptive process requiring concerted effort at a meaningful integration of technology into health [30,31]
- Extends beyond the health sector [24,27,30]
- Person-centered [6,32]
- A transition process [24]

### Digitalization of Public Health

We identified 5 perspectives related to digitalization in the literature (Textbox 2). Overall, 3 articles referred to digitalization in terms of the *integration of technology in the production of services* [24,29,30]. Of note is that 2 articles used the third as a main reference to this claim. Furthermore, these articles suggested that the integration of technology “allows health services to be organized and delivered in new ways” [24,29,30]. Two of the articles further described digitalization as less of a technical process as in digitization and more of an “organizational and cultural process” [29,30]. Another article referred to digitalization as a process that “supports public health” principles and enables their implementation but does not alter the goals of public health [28]. Yet another article described digitalization in terms of the “establishment and ongoing maintenance of basic infrastructure,” including but not limited to hospital information systems, electronic medical records, mobile health, and public health surveillance [31].

### Digital Transformation of Public Health

Regarding digital transformation in relation to public health, we identified 7 perspectives. First, 4 articles described digital transformation in terms of its “complexity and multifaceted dimensions,” requiring interdisciplinary collaborations to ensure the influential process of transformation [6,24,29,30]. Three of the articles described digital transformation as a “fundamental change in the culture and model of delivery” of health services [24,25,30]. Furthermore, 3 articles suggested that digital transformation “extends beyond health care” to other industries [24,27,30]. These 3 articles described digital transformation as being both health-specific and driven by the broader changes in society, including the widespread availability of smartphones and the increased awareness and tracking of health and lifestyle data, as well as storage and processing of large amounts of heterogeneous data that may not be directly related to health but are relevant in understanding health and health outcomes in populations. Digital transformation was also described in 4 articles as a “disruptive process involving concerted effort to meaningfully integrate technologies” and their related new processes in public health services [4,24,27,30]. This involves the formation of nontraditional partnerships across governments, academia, the technology industry, and scientific bodies. In addition, 2 articles asserted that digital transformation of public health services is founded strongly on user needs (ie, it is person-centered) [6,32]. However, 1 article used digitalization and digital transformation interchangeably and described digital transformation as the process of *transition* in which more public health services and processes will be digitalized [24]. Of note, many articles described these processes in relation to health services, health systems, and the health sector, with public health services subsumed within all 3 terms [24,31].

## Discussion

### Principal Findings

In this review, we sought to understand how public health researchers and practitioners conceptualize and define digital public health. Overall, we found that, as this emerging field draws growing attention, the term *digital public health* has been diversely defined. First, we found 2 formal definitions of digital public health. Although the Public Health England definition is predicated on digital transformation and embraces a transformational role for digital technologies in public health [6], the definition by Odone et al [28] suggests that digitalization is essential in a supporting role that helps facilitate existing public health goals. Despite different visions of the role of digital technology in public health, both definitions agree that digital public health involves the integration of digital technologies into public health to achieve public health goals, suggesting that this integration can potentially improve outcomes and efficiency of services.

**Figure 2:**
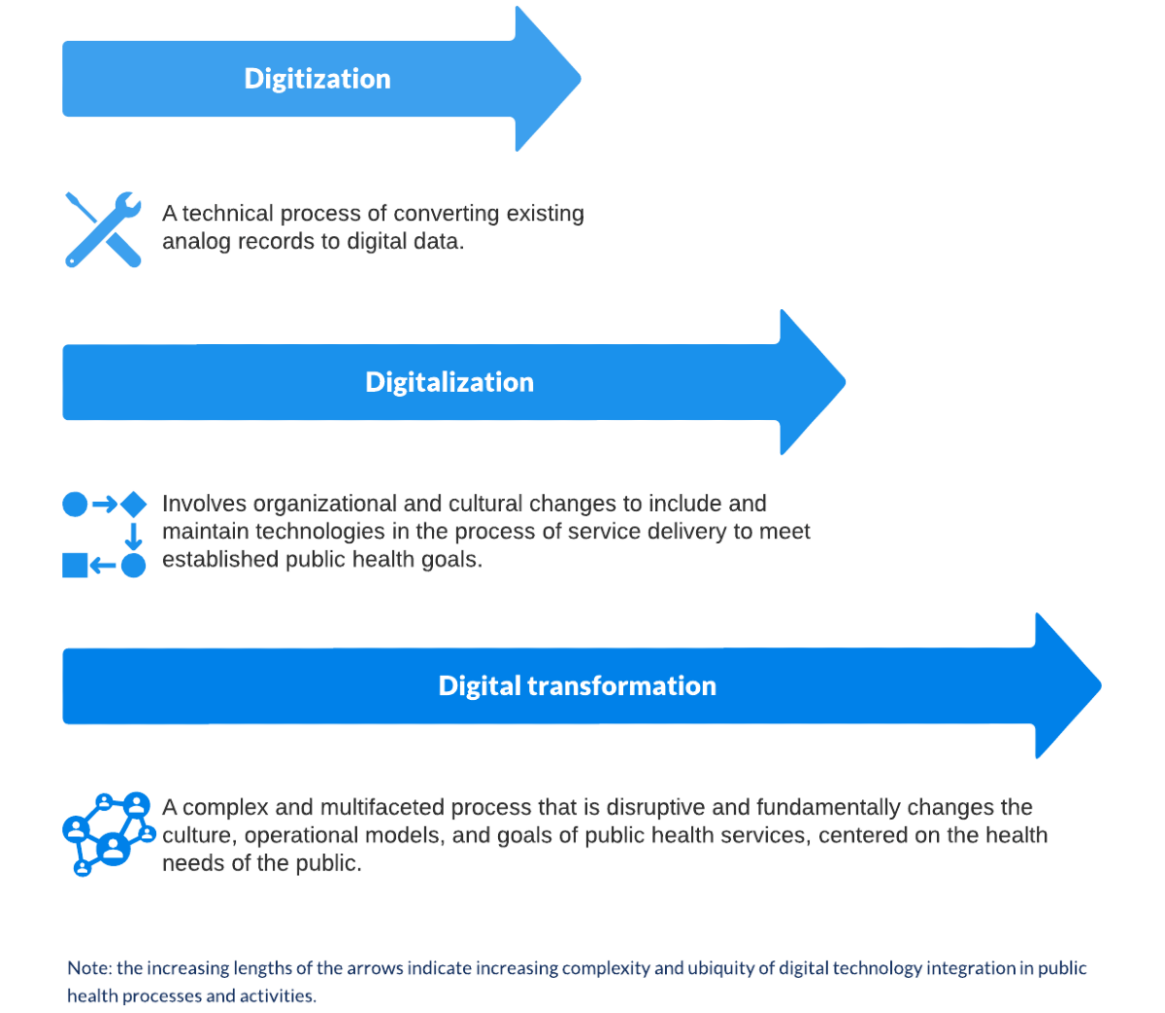
Emergent perspectives on digitization, digitalization, and digital transformation in relation to public health from thematic analysis.

Informal descriptions of the roles played by digitization, digitalization, and digital transformation in relation to public health were identified. Although there were some divergent views, the general perception in the reviewed literature was that all 3 concepts represent increasing levels of complexity, comprehensiveness, and thoughtfulness in the integration of digital technologies into public health practices (Figure 2), where digital transformation encompasses the most complex and fundamental integration of digital technology across the sector [30]. Nevertheless, some authors conceptualized digital transformation as being a transitional phase on the way to the digitalization of public health [24].

### Digitization in Public Health

Our finding of digitization as a technical process involving the conversion of analog information into digital formats (signals) agrees with descriptions of the term in the literature [33-35]. This process is considered the most basic attempt at using digital technologies that allows converted digital information to be used in multiple ways, even beyond the initial use case the data were generated to address [34,36]. For example, digitization of existing paper-based immunization records may allow the data to be linked to other databases to evaluate health prevention programs [34].

### Digitalization in Public Health

In contrast, digitalization has been described in the literature as a sociotechnical process that involves the integration of digital technologies into existing operations and tasks with the goal of improving efficiency and *adding value* to users [34,35]. Such integration of digital technologies into existing operations may allow for data to be automatically generated in a way that enables automation and improved efficiency. An example of digitalization is the use of computer systems and electronic health records at a sexually transmitted infection clinic that allows for the generation of health data during the process of service delivery, ensuring that such data may be used to inform disease prevention, surveillance, and health care quality improvement [37-39]. Nevertheless, it must be acknowledged that digitization and digitalization have been used interchangeably in the literature, with digitization used in this context to refer to processes that are more consistent with digitalization [40]. We make the distinction between digitization and digitalization to draw attention to the higher level of complexity involved in the process of digitalizing public health services [28,30].

### Digital Transformation in Public Health

The descriptions of digital transformation appear to be more consistent in the literature and are congruent with our findings [40]. The general perception is that digital transformation represents the most comprehensive, complex, and pervasive form of technology integration [30,34,35]. More specifically, it is said to involve the development of new *business models* that are more aligned with service user needs (ie, the public) in a way that offers more *value* to the public and the implementing organizations [30,34,35]. For example, in sexually transmitted infection testing services, digital transformation may be envisioned as the creation of web-based testing portals, self-testing models, health education, and promotion of services through mobile apps and clinic-based referral systems that are all interconnected in a technological ecosystem built around the needs of the public to ensure that health is equitable and of high quality. Such pervasive transformation in technologies, management processes, and relationships is said to require development of new competencies among workers within organizations and agencies, with a focus on cross-functional collaborations as opposed to siloed operations within specific health agencies and aspects of the health systems [35,41]. Digital transformation is considered as typically involving a series of distinct digitalization initiatives with an overarching aim to facilitate far-reaching, person-centered organizational change [40]. Furthermore, the depth of digital integration implies that organizations and agencies must fine-tune their key performance indicators [34]. Practitioners suggest that these indicators should extend beyond user access numbers or similar indicators associated with digitalization (eg, the number of people accessing health services on the web or the number of downloads) to more user-centric measures such as web-based sentiment, engagement, and value sharing [34]. The collective assessment of these intermediate measures may describe how well the complex systems of change are operating and how well long-term public health goals are achieved [34].

### Clarifying Digitization, Digitalization, and Digital Transformation in Relation to Public Health

Despite the increasing popularity of the term *digital public health*, our study showed that researchers and practitioners have not yet attempted to achieve conceptual consensus on how it is defined. We also found that, despite general agreements about the implications of digitization, digitalization, and digital transformation of public health, the health-related literature still conflates these terms. For example, a recent call to use the COVID-19 pandemic as a catalyst for digitization in Africa went on to describe processes that might be best described as digitalization [42]. Similarly, a more recent definition of digital public health (the only additional definition we found after the completion of our literature search) suggested that digital public health referred to the use of technology, new types of data, and new ways of working that come with the digitization of public health and associated data [43]. However, the description of these processes might at the minimum be referred to as digitalization. The varied perceptions and conflation of the terms *digitization*, *digitalization*, and *digital transformation* is not new, as these terms have sparked long-standing debates among practitioners and researchers in other industries such as business, finance, and commerce that have also sought to integrate digital technologies [34-36]. Despite this, descriptions of digitization, digitalization, and digital transformation in other fields of research and industry are mostly congruent with our findings, albeit with a few divergent views [34].

Clarifying the definition of digital public health and making the distinction between digitization, digitalization, and digital transformation as applied in this emergent field has significant implications for ongoing research and development. Conceptual clarity can help define goals and operational strategies for integrating digital technologies into public health to ensure their successful implementation and evaluation. Such conceptual clarity may also be helpful in ensuring comparability of outcomes involving digital technologies across jurisdictions. Furthermore, determining the extent to which digital technologies may be integrated into public health services is helpful for advocacy and planning purposes. By this, we mean a careful consideration of the role of digital technologies in facilitating organizational goals, either as a supporter (which does not alter public health goals) or as an enabler (which fundamentally alters the operational models to achieve public health goals) [44].

### The Role of Digitalization in Public Health and Its Implication for Practice

Conceptualizing digital public health in relation to digitalization supposes that digital technologies play a supportive role or serve as *tools* available to public health practitioners to achieve existing public health goals [23,28]. This conceptualization aims to integrate digital technologies to meet public health needs more efficiently while firmly maintaining focus on public health goals rather than on technologies and how they alter public health functions [28]. Health system goals to ensure the quality, accessibility, efficiency, and equity of health services remain unaltered despite acknowledgment that digital technologies must be thoughtfully leveraged in public health efforts [28]. Perhaps envisioning digital public health in this manner resists the technological determinism that has been characteristic of a more pervasive integration of digital technologies [45,46]. Technological determinism is the assertion that the use of digital technologies inevitably leads to improved processes, services, and outcomes [45]. This assertion is as yet unproven. However, conceptualizing digital public health as a supporting tool to improve existing public health functions may result in siloed, heterogeneous digitalization initiatives with potentially limited interoperability and impact on the public and health systems. This limitation has already been seen in the existence of multiple pilot digital initiatives with data systems that are not interoperable within mainstream public health systems [47].

### The Role of Digital Transformation in Public Health and its Implication for Practice

Conversely, conceptualizing digital public health as a product of digital transformation may offer a few advantages. In addition to potentially being more person-centered and cost-efficient [6], a comprehensive and structured integration of digital technologies into public health functions would possibly allow public health practitioners and decision-makers to consider ways to ensure a cohesive approach to digital public health that transcends current public health silos. This approach embraces the cross-functional, nontraditional relationships between professionals within and outside traditional public health practice, which allows for the exchange of information across public health systems and other health-related systems to gain a better understanding of the determinants of health and identify strategies to improve health and achieve public health goals and functions. This contrasts with siloed approaches to integrate digital technologies within organizations, health agencies, and health systems that have often resulted in interoperability issues, inhibiting the development of digital public health for years [48]. Nevertheless, public health practitioners, managers, and decision-makers would also face significant challenges. The major issue would be maintaining fundamental public health goals like health equity if public health systems are to be fundamentally changed despite the existing digital divide [49]. This is important given that access to digital health technologies is often structured by peoples’ socioeconomic status and social positions [1,49-51]. This differential access to technology is also often reflected as over- and underrepresentation of various sociodemographic groups in the digital data informing public health practice [52]. If cross-functional collaborations are to be fostered, further challenges exist regarding data ownership, privacy, and security, as well as clarifying the roles of public and private partners in digital public health [48].

There are internal and external pressures to envision digital public health as a product of digital transformation [47,53]. As digital change occurs outside of health care, public sentiments now suggest that people must be considered as informed health users who demand involvement in decisions and actions concerning their health [47,54]. Increases in health care costs associated with aging populations provides further incentives to empower users of public health services through in-depth integration of digital technologies [47]. Finally, the growing omnipresence of user-generated data from activities outside the public health systems further creates pressure to conceptualize digital public health as a digital transformation that ensures integration into a well-coordinated health system [34].

### Future Directions

We do not intend to make assertions as to which conceptualization of digital public health, as related to digitalization and digital transformation, is better suited for the complex challenges facing public health practitioners. Rather, we present the implications of each definition of digital public health as identified in the literature. Further qualitative research is required to delineate meanings that researchers and practitioners ascribe to digital public health on the basis of geopolitical jurisdictions [28,30]. Our study suggests that Europe-based scholars are leading in the conceptualizations of digital public health, at least within the academic and health systems literature, compared with discussions taking place in English in other continents. As part of our work, we intend to consult with health agencies, public health practitioners, and other relevant experts to adopt a working definition and conceptual framework for digital public health within our context. Research is also required to evaluate which conceptualization better promotes public health goals and facilitates integration of digital technologies that improve the health and health-related outcomes of the public. As interest in digital public health continues to grow during the COVID-19 pandemic, it is imperative to generate robust and meaningful evidence to clearly guide the development of the field. Given the diverse definitions that digital health has attained, consensus building around the envisioning, definition, and operationalization of digital public health is critical.

### Limitations

The findings of this study should be considered in view of its limitations. First, our literature search was conducted in June 2020. We are aware of the sharp increase in publications on digital health and digital public health as a result of the COVID-19 pandemic. Given that more recent literature has been skewed by attention to the COVID-19 pandemic, we considered it more expedient to assess articles published by the date of our initial search in 2020. To ensure that we did not miss any significant additions, we conducted a cursory search of *digital public health* on PubMed in March 2021 and found only one additional definition published in English by Murray et al [43] that was referenced in the discussion above. Furthermore, our restriction to articles published in English may have inadvertently excluded other potential definitions of digital public health.

### Conclusions

Digital public health continues to be diversely defined and conceptualized in the literature as attention to the subject increases among researchers and practitioners. Available definitions are divergent in relation to their conceptualization of the roles of digitalization and digital transformation in digital public health. It is still unclear which definition would better help improve public health practices and outcomes. Public health researchers and practitioners can better develop the field with more clarity and consensus on the definition of digital public health, and the role of digitalization and digital transformation in this definition, by encapsulating the intent of their practice and providing a clear road map for ongoing development.

## Appendix

Multimedia Appendix 1 Agency and country websites searched for gray literature.

Multimedia Appendix 2 Framework for full-text review.

## References

[1] Brewer LC, Fortuna KL, Jones C, Walker R, Hayes SN, Patten CA, Cooper LA (2020). Back to the future: achieving health equity through health informatics and digital health. JMIR Mhealth Uhealth.

[2] World Health Organization (2019). WHO Guideline: Recommendations on Digital Interventions for Health Systems Strengthening.

[3] Crawford A, Serhal E (2020). Digital health equity and COVID-19: the innovation curve cannot reinforce the social gradient of health. J Med Internet Res.

[4] (2020). Draft Global Strategy on Digital Health 2020-2025. World Health Organization (WHO).

[5] Azzopardi-Muscat N, Sørensen K (2019). Towards an equitable digital public health era: promoting equity through a health literacy perspective. Eur J Public Health.

[6] (2017). Digital-first public health: public health england's digital strategy. Public Health England.

[7] Dual degree programs in public health data science. McGill University.

[8] (2021). UCL IRDR Centre for Digital Public Health in Emergencies (dPHE). UCL Institute Risk Disaster Reduction.

[9] (2021). Call for papers: digital public health. BMC.

[10] Digital Public Health. Frontiers in Public Health.

[11] (2019). The 9th International Digital Public Health Conference.

[12] Porta M, Last JM (2018). A Dictionary of Public Health (2nd ed.).

[13] Cohen AB, Dorsey ER, Mathews SC, Bates DW, Safavi K (2020). A digital health industry cohort across the health continuum. NPJ Digit Med.

[14] Budd J, Miller BS, Manning EM, Lampos V, Zhuang M, Edelstein M, Rees G, Emery VC, Stevens MM, Keegan N, Short MJ, Pillay D, Manley E, Cox IJ, Heymann D, Johnson AM, McKendry RA (2020). Digital technologies in the public-health response to COVID-19. Nat Med.

[15] Iyamu I, Gómez-Ramírez O, Xu AXT, Chang H-J, Haag D, Watt S, Gilbert M (2021). Defining the scope of digital public health and its implications for policy, practice, and research: protocol for a scoping review. JMIR Res Protoc.

[16] Arksey H, O'Malley L (2005). Scoping studies: towards a methodological framework. Int J Soc Res Methodol.

[17] Levac D, Colquhoun H, O'Brien KK (2010). Scoping studies: advancing the methodology. Implement Sci.

[18] Tricco AC, Lillie E, Zarin W, O'Brien KK, Colquhoun H, Levac D, Moher D, Peters MDJ, Horsley T, Weeks L, Hempel S, Akl EA, Chang C, McGowan J, Stewart L, Hartling L, Aldcroft A, Wilson MG, Garritty C, Lewin S, Godfrey CM, Macdonald MT, Langlois EV, Soares-Weiser K, Moriarty J, Clifford T, Tunçalp Ö, Straus SE (2018). PRISMA Extension for Scoping Reviews (PRISMA-ScR): checklist and explanation. Ann Intern Med.

[19] (2017). Public health: a conceptual framework. Canadian Public Health Association.

[20] Government of Canada.

[21] Better systematic review management. Covidence.

[22] Braun V, Clarke V (2006). Using thematic analysis in psychology. Qual Res Psychol.

[23] Azzopardi-Muscat N, Ricciardi W, Odone A, Buttigieg S, Paget DZ (2019). Digitalization: potentials and pitfalls from a public health perspective. Eur J Public Health.

[24] Expert Panel on effective ways of investing in Health (EXPH) (2018). Assessing the Impact of Digital Transformation of Health Services.

[25] Fitzpatrick F, Doherty A, Lacey G (2020). Using artificial intelligence in infection prevention. Curr Treat Options Infect Dis.

[26] Lu Z (2009). Information technology in pharmacovigilance: benefits, challenges, and future directions from industry perspectives. Drug Healthc Patient Saf.

[27] Mählmann L, Reumann M, Evangelatos N, Brand A (2017). Big data for public health policy-making: policy empowerment. Public Health Genomics.

[28] Odone A, Buttigieg S, Ricciardi W, Azzopardi-Muscat N, Staines A (2019). Public health digitalization in Europe. Eur J Public Health.

[29] Rachadell J, Brinzac MG (2020). Digital Health in 2019. A summary report of the track of digital health at the 12th European Public Health conference 2019 in Marseille, France. https://eupha.org/repository/conference/2019/Digital_Health_in_2019_V2.pdf.

[30] Ricciardi W, Pita Barros P, Bourek A, Brouwer W, Kelsey T, Lehtonen L, Expert Panel on Effective Ways of Investing in Health (EXPH) (2019). How to govern the digital transformation of health services. Eur J Public Health.

[31] (2018). Towards a roadmap for the digitalization of national health systems in Europe. World Health Organization (WHO) Regional Office for Europe.

[32] (2016). The A-Z of digital public health. GOV.UK.

[33] Gange SJ, Golub ET (2016). From smallpox to big data: the next 100 years of epidemiologic methods. Am J Epidemiol.

[34] Verhoef PC, Broekhuizen T, Bart Y, Bhattacharya A, Qi Dong J, Fabian N, Haenlein M (2021). Digital transformation: a multidisciplinary reflection and research agenda. J Bus Res.

[35] Saarikko T, Westergren UH, Blomquist T (2020). Digital transformation: five recommendations for the digitally conscious firm. Bus Horiz.

[36] Gobble MM (2018). Digitalization, digitization, and innovation. Res Technol Manag.

[37] Gilbert M, Salway T, Haag D, Fairley CK, Wong J, Grennan T, Uddin Z, Buchner CS, Wong T, Krajden M, Tyndall M, Shoveller J, Ogilvie G (2017). Use of GetCheckedOnline, a comprehensive web-based testing service for sexually transmitted and blood-borne infections. J Med Internet Res.

[38] Knight R, Karamouzian M, Salway T, Gilbert M, Shoveller J (2017). Online interventions to address HIV and other sexually transmitted and blood-borne infections among young gay, bisexual and other men who have sex with men: a systematic review. J Int AIDS Soc.

[39] Gilbert M, Haag D, Hottes TS, Bondyra M, Elliot E, Chabot C, Farrell J, Bonnell A, Kopp S, Andruschak J, Shoveller J, Ogilvie G (2016). Get checked...where? The development of a comprehensive, integrated internet-based testing program for sexually transmitted and blood-borne infections in British Columbia, Canada. JMIR Res Protoc.

[40] (2018). Digitization, digitalization, and digital transformation: confuse them at your peril. Forbes.

[41] Verina N, Titko J (2019). Digital transformation: conceptual framework. Proceedings of 6th International Scientific Conference on Contemporary Issues in Business, Management and Economic Enginering' 2019.

[42] Bensbih S, Essangri H, Souadka A (2020). The Covid19 outbreak: a catalyst for digitization in African countries. J Egypt Public Health Assoc.

[43] Murray CJ, Alamro NM, Hwang H, Lee U (2020). Digital public health and COVID-19. Lancet Public Health.

[44] Hess T, Matt C, Benlian A, Wiesböck F (2016). Options for formulating a digital transformation strategy. MIS Q Exec.

[45] McIntyre D (2003). Technological determinism: a social process with some implications for ambulance paramedics. Aus J Paramed.

[46] Gómez-Ramírez O, Iyamu I, Ablona A, Watt S, Xu AXT, Chang H, Gilbert M (2021). On the imperative of thinking through the ethical, health equity, and social justice possibilities and limits of digital technologies in public health. Can J Public Health.

[47] Maldaner N, Tomkins-Lane C, Desai A, Zygourakis CC, Weyerbrock A, Gautschi OP, Stienen MN (2020). Digital transformation in spine research and outcome assessment. Spine J.

[48] M Bublitz F, Oetomo A, S Sahu K, Kuang A, X Fadrique L, E Velmovitsky P, M Nobrega R, P Morita P (2019). Disruptive technologies for environment and health research: an overview of artificial intelligence, blockchain, and internet of things. Int J Environ Res Public Health.

[49] Rodriguez JA, Clark CR, Bates DW (2020). Digital health equity as a necessity in the 21st century cures act era. JAMA.

[50] Sinha C, Schryer-Roy A-M (2018). Digital health, gender and health equity: invisible imperatives. J Public Health (Oxf).

[51] Hargittai E, Hinnant A (2008). Digital inequality: differences in young adults' use of the internet. Commun Res.

[52] Lee EWJ, Viswanath K (2020). Big data in context: addressing the twin perils of data absenteeism and chauvinism in the context of health disparities research. J Med Internet Res.

[53] Mergel I, Edelmann N, Haug N (2019). Defining digital transformation: results from expert interviews. Gov Inf Q.

[54] Hunt D, Koteyko N, Gunter B (2015). UK policy on social networking sites and online health: from informed patient to informed consumer?. Digit Health.

