# Supplementary material for: Defining Digital Public Health and the Role of Digitization, Digitalization, and Digital Transformation: Scoping Review
Source: JMIR Public Health Surveill. 2021 Nov 26;7(11):e30399. doi: 10.2196/30399 (PMC8665390; doi:10.2196/30399)
Supplement: Multimedia Appendix 1 [file publichealth_v7i11e30399_app1.docx]

# **Appendix 1: Agency/Country Websites searched for grey literature**

| **Country/Jurisdiction** | **Agency** |
| --- | --- |
| Intergovernmental | World Health Organization |
| Europe | European Public Health Association |
| Australia | Public Health Association of Australia, Government of Canada |
| Canada | Government of Canada  Public Health Agency of Canada  Canadian Institutes of Health Research  Canadian Public Health Association  National Collaborating Centres for Public Health  National Collaborating Centres for Determinants of Health  Canadian Agency for Drugs and Technologies in Health |
| United Stated of America | US Centers for Disease Control and Prevention  American Public Health Association |
| United Kingdom | UK Public Health Association  National Health Service |
